# Auricular Electroacupuncture Reduced Inflammation-Related Epilepsy Accompanied by Altered TRPA1, pPKC*α*, pPKC*ε*, and pERk1/2 Signaling Pathways in Kainic Acid-Treated Rats

**DOI:** 10.1155/2014/493480

**Published:** 2014-07-24

**Authors:** Yi-Wen Lin, Ching-Liang Hsieh

**Affiliations:** ^1^Graduate Institute of Acupuncture Science, College of Chinese Medicine, China Medical University, Taichung 40402, Taiwan; ^2^Research Center for Chinese Medicine & Acupuncture, China Medical University, Taichung 40402, Taiwan; ^3^Graduate Institute of Integrative Medicine, College of Chinese Medicine, China Medical University, 91 Hsueh-Shih Road, Taichung 40402, Taiwan; ^4^Department of Chinese Medicine, China Medical University Hospital, Taichung 40402, Taiwan

## Abstract

*Background*. Inflammation is often considered to play a crucial role in epilepsy by affecting iron status and metabolism. In this study, we investigated the curative effect of auricular acupuncture and somatic acupuncture on kainic acid- (KA-) induced epilepsy in rats. *Methods*. We established an epileptic seizure model in rats by KA (12 mg, ip). The 2 Hz electroacupuncture (EA) was applied at auricular and applied at *Zusanli* and *Shangjuxu* (ST36-ST37) acupoints for 20 min for 3 days/week for 6 weeks beginning on the day following the KA injection. *Results*. The electrophysiological results indicated that neuron overexcitation occurred in the KA-treated rats. This phenomenon could be reversed among either the auricular EA or ST36-ST37 EA treatment, but not in the sham-control rats. The Western blot results revealed that TRPA1, but not TRPV4, was upregulated by injecting KA and could be attenuated by administering auricular or ST36-ST37 EA, but not in the sham group. In addition, potentiation of TRPA1 was accompanied by increased PKC*α* and reduced PKC*ε*. Furthermore, pERK1/2, which is indicated in inflammation, was also increased by KA. Furthermore, the aforementioned mechanisms could be reversed by administering auricular EA and could be partially reversed by ST36-ST37 EA. *Conclusions*. These results indicate a novel mechanism for treating inflammation-associated epilepsy and can be translated into clinical therapy.

## 1. Introduction

Epilepsy is a common clinical neurological disease, with a prevalence of approximately 1%, that is highly associated with inflammation and oxidative stress [1–4]. Epilepsy is characterized by uncontrolled discharges caused by neuronal hyperactivity in the temporal lobe and the hippocampus. Neural and cognitional functions are lost during a seizure. Glutamate is a major excitatory neurotransmitter in mammalian brains and an agonist of the KA subtype of glutamate receptors, which is typically used to induce epilepsy in both rats and mice [5–7]. KA-induced epilepsy symptoms are extremely similar to temporal lobe seizures that occur in humans [8–10]. Glutamate receptor-dependent overactivation or the lack of *γ*-aminobutyric acid (GABA) receptor-dependent inhibition causes central nervous system discharge, which is principally responsible for epilepsy [11, 12]. Several antiepileptic drugs, such as topiramate [13] and gabapentin [14], inhibit excitatory glutamate receptors and the activation of inhibitory GABA receptors. However, more than 30% of patients who use traditional antiepileptic drugs experience uncontrolled seizures and side effects in clinics [15]. Accordingly, developing effective antiepileptic drugs is crucial and urgent.

Recently, controlling body temperature, particularly by inducing hypothermia, has been suggested to reduce seizure activity [16, 17]. Lomber et al. reported that cooling body temperature can effectively inhibit synaptic transmission and reduce spontaneous epileptiform discharge [18]. Hypothermia is frequently used as a standard treatment for stroke, brain injury, and uncontrolled epilepsy. However, the specific function of cooling the brain and the underlying mechanisms remain unclear. Transient receptor potential ankyrin 1 (TRPA1), which belongs to the TRPA (ankyrin) family, has been reported to detect noxious thermal pain [19, 20]. TRPA1 is reported to mediate the inflammatory actions of environmental irritants and proalgesic agents [21]. TRPA1 is a calcium-permeable ion channel expressed in peripheral nociceptive neurons [22, 23] and the brain [24]. TRPA1 activity can be regulated by protein kinase A (PKA), protein kinase C (PKC), and extracellular signal-regulated kinase (ERK) in inflammatory and neuropathic pain models [25, 26]. TRPV4 is associated with osmotic pressure and mechanical and thermal sensitivity that is expressed in heterologous systems [27, 28]. Mice without TRPV4 receptor cannot regulate the serum osmolarity and are insensitive to noxious stimuli [29, 30]. TRPV4 was involved in several kinds of pain mediation including pain from mechanical hyperalgesia, complications of vincristine chemotherapy, diabetes, alcoholism, and acquired immune deficiency syndrome therapy [31, 32]. TRPV4 is indicated to be participating in epilepsy regulation [33].

Recently, several PKC isoforms (PKC*α*, PKC*β*, PKC*δ*, PKC*ε*, PKC*η*, and PKC*ζ*) were reported to participate in epilepsy [34, 35]. Liu et al. reported that, in a pilocarpine epilepsy model, PKC*δ*, PKC*η*, and PKC*ζ* were decreased in epileptic hippocampi. By contrast, PKC*α*, PKC*β*1, and PKC*ε* increased in epileptic mice. In addition, Tang et al. suggested that PKC*δ* increased in the hippocampus in pilocarpine-induced epilepsy. However, PKC*γ* and PKC*ε* were attenuated in an epileptic hippocampus. After the epileptic time was extended, PKC*β*1, PKC*δ*, PKC*η*, and PKC*ζ* were potentiated at 7 and 31 days after pilocarpine injection [26]. Extracellular signal-regulated kinase 1/2 (ERK1/2) has been reported to be activated in neurons, particularly in people with epilepsy. Eun et al. indicated that PKC*α* is involved in microglial activation process under pathological conditions including neuroinflammation and neurodegeneration [36]. During KA-induced epileptiform seizures, pERK1/2 activation is highly associated with neuroprotection [37].

Acupuncture has been used for over 3000 years and has been based on the traditional Chinese medicine theory. The analgesic effect of acupuncture is widely accepted already. Recently, acupuncture is also well established for epilepsy treatment. Liu and coauthors indicated stimulation of the ear acupuncture for the treatment of epilepsy [38]. Auricular vagus nerve stimulation is also reported to serve as an alternative therapy for drug-resistant epilepsy [39]. Acupuncture is highly regarded as a crucial therapy in inhibiting KA-induced hippocampal cell death and inflammatory events in mice [40–42].

In this study, we investigated the crucial effects and detailed cellular mechanisms of acupuncture by using an electrophysiology technique. Epileptic discharge was increased in the hippocampal CA1 region of KA-injected rats and further reduced by administering auricular and somatic acupuncture. TRPA1, but not TRPV4, protein levels were potentiated in the KA-treated rats. The pPKC*α* and pERK1/2 protein levels were increased; however, pPKC*ε* proteins were reduced in the KA group. All of the aforementioned phenomena were reversed by administering auricular acupuncture. This novel finding indicates that auricular EA is maybe used for clinical epileptic therapy.

## 2. Materials and Methods

### 2.1. Animals

A total of 30 male Sprague-Dawley (SD) rats weighing 200–300 g were used in this study. The Institute of Animal Care and Use Committee of China Medical University approved the use of these animals (permit No. 101-116-N). In addition, the* Guide for the Use of Laboratory Animals* (National Academy Press) was followed.

### 2.2. Establishing an Epileptic Seizure Model

A total of 30 SD rats were used in the experiments. Four days before the electroencephalogram (EEG) and electromyogram (EMG) recordings were conducted, all of the rats underwent stereotactic surgery. The scalp was then incised from the midline to expose the skull. Stainless steel screw electrodes were implanted on the dura above the bilateral sensorimotor cortices to function as recording electrodes. A reference electrode was placed in the frontal sinus. Bipolar electrical wires were placed on the neck muscles for the EMG recordings. Electrodes were connected to an EEG- and EMG-monitoring machine (MPlOOWSW, BIOPAC Systems, Inc., CA, USA). The epileptic seizures were captured using a video recording epileptic behavioral analysis system (SeizureScan, Clever Sys., Inc., Virgina, USA) that occurred in the rats were confirmed by observing behavior (e.g., wet-dog shakes, paw tremors, and facial myoclonia under a freely moving and conscious state) and epileptiform discharges on EEG recordings. The rats were randomly divided into 5 experimental groups on which electrophysiological studies (15 rats in total and 3 rats in each group) and Western blot analysis of TRPA1, TRPV4, PKC*α*, PKC*ε*, and pERK1/2 (15 rats in total and 3 rats per group) were performed after the rats experienced KA-induced epileptic seizures. Each experiment was divided into 5 groups: (1) a control group, in which the rats were injected with phosphate buffer solution (PBS) ip only; (2) the KA group, in which the rats were injected with 12 mg/kg ip of KA only; (3) the auricular group, in which the rats received 2 Hz EA (anion placed at the ear apex and cathode placed at the ear lobe by using a clip electrode at a frequency of 2 Hz; the stimulus intensity, indicated by a visual ear twitch, was maintained for 20 min and 10 min for the left and right ears, resp.) for 3 days/week for 6 weeks beginning on the day following the KA injection; (4) the ST36-ST37 group, in which the rats received 2 Hz EA at the Zusanli and Shangjuxu (ST36-ST37) acupoints (anode placed at ST36 and cathode placed at ST37 by inserting 2 stainless steel acupuncture needles into the muscle layer) for 3 days/week for 6 weeks beginning on the day following the KA injection; and (5) the sham group, for which the methods used were identical to those used for the ST36-ST37 group; however, the needles penetrated the ST36-ST37 acupoints cutaneously without electric discharge for 3 days/week for 6 weeks beginning on the next day following the KA injection. All of the rats were sacrificed 6 weeks after the KA injection, and the hippocampi were removed for electrophysiological and Western blot studies.

### 2.3. Electrophysiology

Transverse hippocampal slices (450 *μ*m thick) were cut with a vibrating tissue slicer (Campden Instruments, Loughborough, UK) and transferred to an interface-type holding chamber at room temperature. The slices were recovered for at least 120 min and then were transferred to an immersion-type recording chamber, perfused at 2 mL/min. We use the artificial CSF (ACSF) containing the following (mM): 119 NaCl, 2.5 KCl, 26.2 NaHCO_3_, 1 NaH_2_PO_4_, 1.3 MgSO_4_, 2.5 CaCl_2_, and 11 glucose (the pH was adjusted to 7.4 by gassing with 5% CO_2_-95% O_2_). Regarding extracellular recording, a glass pipette filled with 3 M NaCl was placed in the stratum radiatum of the CAl area to record field excitatory postsynaptic potentials (fEPSPs). Bipolar stainless steel electrodes (Frederick Haer Company, Bowdoinham, ME, USA) were placed in the stratum radiatum to stimulate the Schaffer collateral input. Low Mg^2+^ artificial cerebrospinal fluid solution was used to elicit epileptiform spikes. Regarding whole-cell patch recording, a pipette was filled with the following chemicals (in mM): 116 potassium gluconate, 6 KCl, 2 NaCl, 30 HEPES, 0.5 EGTA, 4 Mg-ATP, and 0.3 Na-GTP (the pH was adjusted to 7.2 by using KOH, and the osmolarity was adjusted to 300 mOsm). The whole-cell patch recordings were performed on the CA1 pyramidal cells by using a visualized patch technique. An Axopatch-700B amplifier (Axon Instruments, Inc., Foster City, CA, USA) was switched to current-clamp mode once the whole-cell recordings were obtained, and the membrane potential was maintained at −65 mV. Recording was terminated and the data were discarded if the serial resistance or input resistance varied by more than 30%. All of the signals were filtered at 2 kHz by using a low-pass Bessel filter included in the Axopatch-700B amplifier and digitized at 5 kHz by using a CED Micro 1401 interface that ran Signal software (Cambridge Electronic Design, Cambridge, UK).

### 2.4. Western Blot Analysis

Bilateral hippocampal proteins were extracted at 6 weeks after the experiments. Total proteins were prepared by homogenizing neurons in a lysis buffer containing 50 mM Tris-HCl with pH 7.4, 250 mM NaCl, 1% NP-40, 5 mM EDTA, 50 mM NaF, 1 mM Na_3_VO_4_, 0.02% NaN_3_, and 1x protease inhibitor cocktail (Amresco, Solon, OH, USA). The extracted proteins (30 *μ*g/sample assessed using the BCA protein assay) were subjected to 8% SDS-Tris glycine gel electrophoresis and transferred to a PVDF membrane. The membrane was blocked using 5% nonfat milk in a TBS-T buffer (10 mM Tris, pH 7.5, 100 mM NaCl, and 0.1% Tween 20), incubated with anti-TRPA1 (1 : 1000, Alomone Labs, Jerusalem, Israel), TRPV4 (1 : 1000, Alomone Labs), PKC*α* (pSer657) (1 : 1000, Millipore, Billerica, MA, USA), PKC*ε* (1 : 500, Novus Biologicals, Littleton, CO, USA), and pERK1/2 (pThr202, pTyr204) (1 : 500, Novus Biologicals, Littleton, CO, USA) in TBS-T containing 1% bovine serum albumin, and incubated for 1 hour at room temperature. Peroxidase-conjugated anti-rabbit and anti-mouse antibodies (1 : 5000, Jackson ImmunoResearch Laboratories, West Grove, PA, USA) were used as secondary antibodies. The bands were visualized using an enhanced chemiluminescencent substrate kit (Thermo Scientific, Waltham, MA, USA) with LAS-3000 Fujifilm (Fuji Photo Film Co. Ltd., Minato, Tokyo, Japan). Where applicable, the image intensities of specific bands were quantified using NIH ImageJ software (Bethesda, MD, USA).

### 2.5. Statistical Analysis

All of the statistical data are presented as the mean ± standard error. Statistical significance between each group was tested using analysis of variance, followed by a post hoc Tukey's test (*P* < .05 was considered statistically significant).

## 3. Results

### 3.1. KA-Induced Epileptic Seizures and EEG Changes

**Figure 1:**
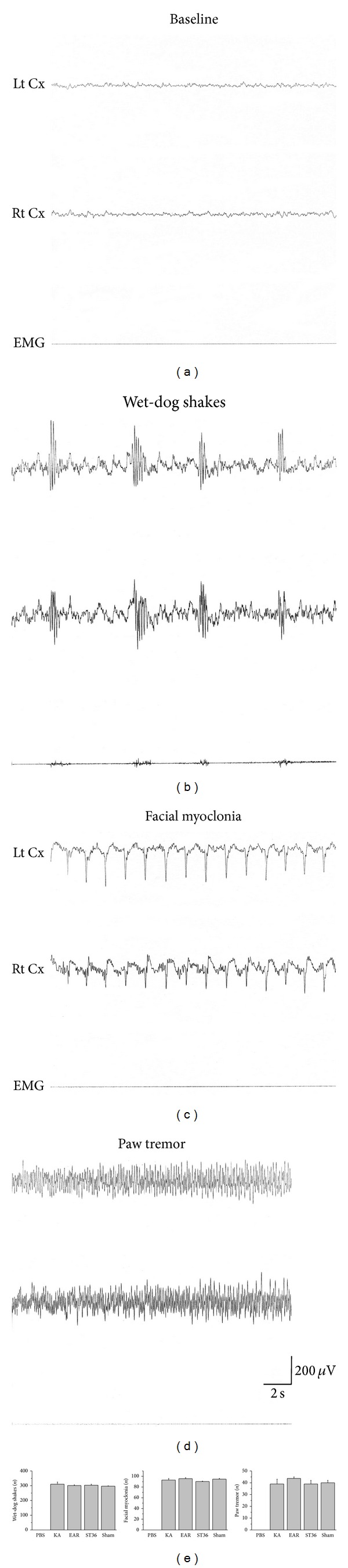
Establishment of epilepsy rats was monitored by electroencephalographic (EEG) signals. Baseline EEG activity in the sensorimotor cortex was characterized by 6–8 Hz activity in rats when awake (a). KA-induced temporal lobe seizures, including wet-dog shakes (WDS) with intermittent polyspike-like activity (b), facial myoclonia with continuous sharp waves (c), and paw tremor (PT) with continuous spike activity (d). All signals were counted and displayed as bar chart (e). Each type of seizure had its own characteristic EEG activity. Lt Cx = EEG recording of the left sensorimotor cortex; Rt Cx = EEG recording of the right sensorimotor cortex; EMG = EMG recording of the neck muscle.

Epilepsy is a major neurological disease caused by the hyperexcitation of neuronal networks in mammalian brains. In this study, we injected rats with KA ip to induce epileptic seizures (30 SD rats; 12 mg/kg per rat). We monitored 3 major types of seizure by observing EEG recordings. We counted wet-dog shakes, paw tremors, and facial myoclonia to ensure the creation of a successfully induced seizure model.  Figure 1(a) shows the baseline recordings. Wet-dog shakes were indicated by intermittent polyspike-like EEG activity (Figure 1(b)). Facial myoclonia was characterized by sharp continual EEG activity (Figure 1(c)). Paw tremors were indicated by continual spikes in EEG activity (Figure 1(d)). Based on these parameters, we used the rats that exhibited seizures with similar degrees of severity for each experiment to investigate the effect of EA on epilepsy (Figure 1(e)).

### 3.2. Epileptic Spikes, Synaptic Transmission, and Paired-Pulse Facilitation in KA-Injected Rats

**Figure 2:**
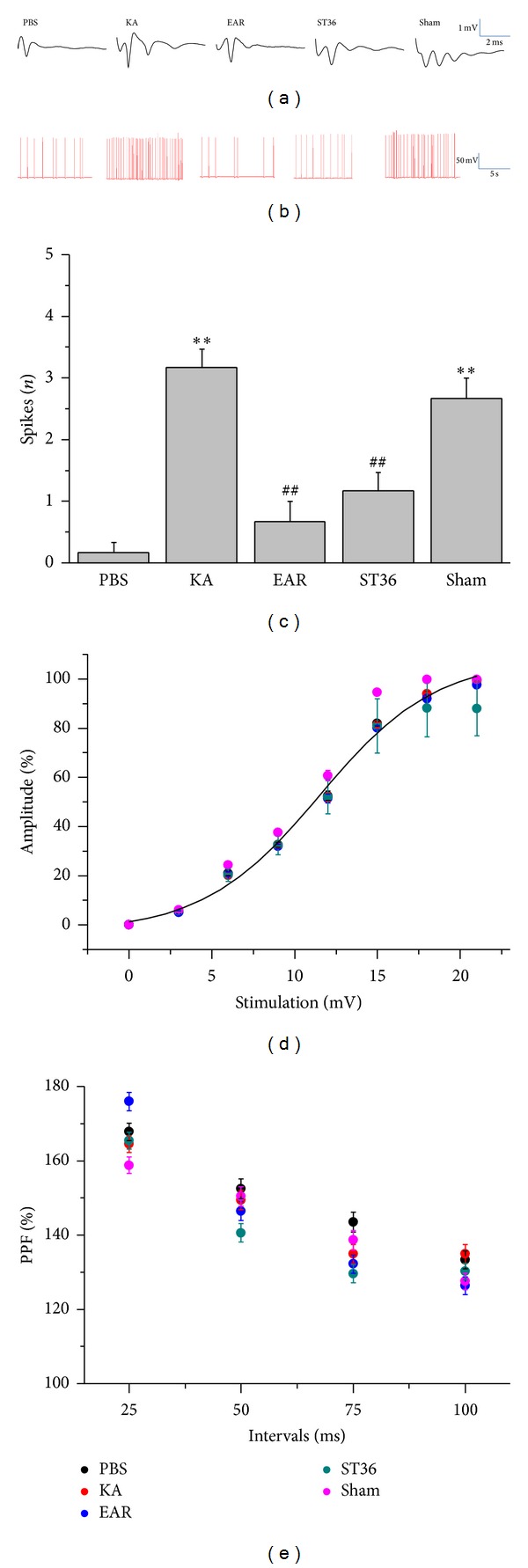
Electrophysiological recording from PBS, KA, EAR (auricular), ST36 (ST36-ST37), and sham-operated rats. Extracellular recordings from PBS, KA, EAR, ST36, and sham groups (a). Whole-cell recording (b). Statistical analysis from each group (c). Input/output ratio from each group (d). Paired-pulses facilitation from each group. *P < .05 compared with PBS group. ^#^ *P* < .05 compared with KA group.

We subsequently verified changes in the CA1 synaptic function in the hippocampal slices from each group. We first used field recordings to recognize fEPSP to measure excitatory spikes. In the hippocampal CA1 areas of the rats in the PBS group, rare spikes after fEPSP occurred (0.17 ± 0.17, *n* = 6 from 3 rats) (Figure 2(a)). Regarding KA-treated rats, several spikes appeared, suggesting hyperexcitability in the epileptic rats (3.17 ± 0.31, *P* < .05 compared with PBS group, *n* = 6 from 3 rats) (Figure 2(a)). The hyperactivity was further attenuated by administering 2 Hz auricular and ST36-ST37 EA (0.67 ± 0.33 and 1.17 ± 0.31 compared with KA group, *n* = 6 from 3 rats, resp.), but not in the sham-operated group (2.67 ± 0.33, *n* = 6 from 3 rats) (Figure 2(a)). Similar results were obtained from the whole-cell patch recordings, as shown in Figure 2(b). The population spikes were statistically analyzed and are illustrated in Figure 2(c). We then examined synaptic transmission by using input-output ratio curves to identify the relationship between fEPSP response and stimulation strength. The fEPSP amplitudes increased as stimulation voltage increased. No significant differences were observed among the groups, indicating similar synaptic transmission among the rats in each group (Figure 2(d)). We further tested whether epileptic discharge could alter presynaptic activity by assessing paired-pulse facilitation. The results indicated that the presynaptic activity was similar among all of the groups (Figure 2(e)). The aforementioned data revealed that neuronal hyperexcitability was altered during an epileptic seizure without changing the basal transmission and presynaptic properties.

### 3.3. TRPA1 Expression Was Altered in KA-Induced Epileptic Seizures and Further Changed by 2 Hz EA at the Auricular and at ST36-ST37 Acupoints

**Figure 3:**
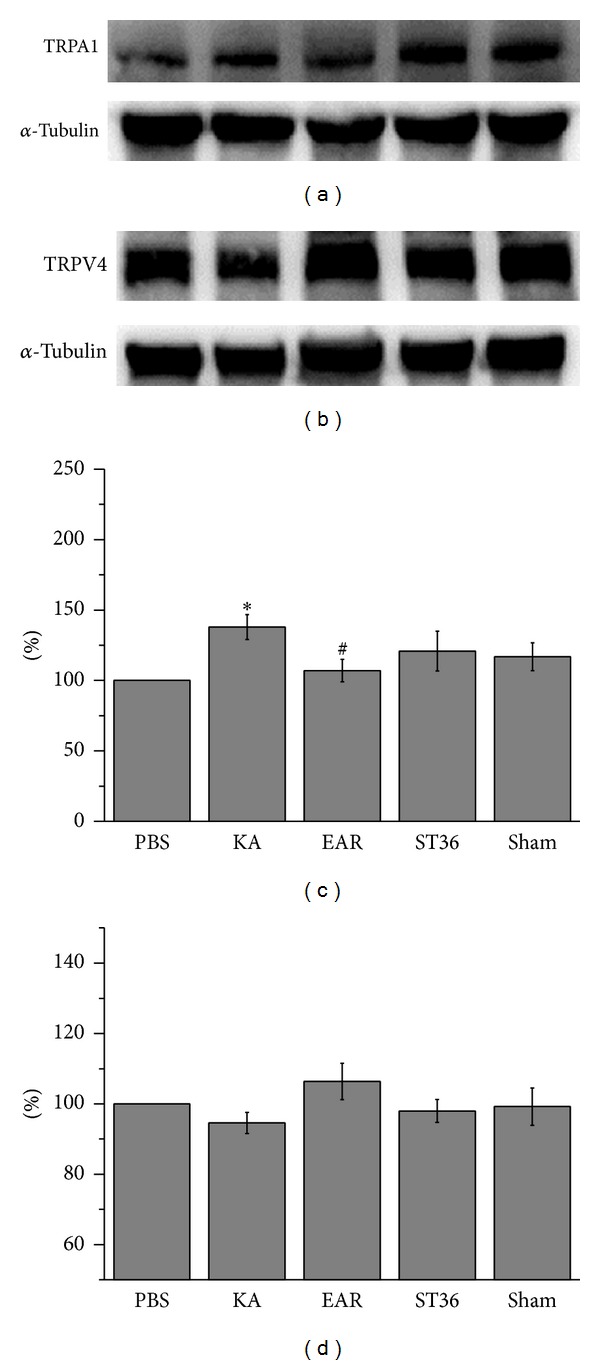
TRPA1 and TRPV4 protein levels. Hippocampus lysates were immunoreacted with specific TRPA1 (a) and TRPV4 (b) antibodies. TRPA1, but not TRPV4, increased with KA injection as compared with the PBS group. TRPA1 protein levels were attenuated by electroacupuncture (EA) at EAR (auricular) as compared with the KA-induced groups. Serious results were not observed in ST36 (ST36-ST37) and sham groups. All statistic results were analyzed and plotted as bar chart in (c) and (d).

According to in vitro and in vivo epilepsy models, controlling body temperature facilitates the reduction of epileptiform discharges. Therefore, we examined thermal receptors in the hippocampal neurons, TRPA1 and TRPV4. The results indicated that TRPA1 protein levels increased after the KA injection, suggesting that upregulation caused by epileptogenesis occurred in the hippocampus of the rats (137.9% ± 8.8%, *P* < .05 compared with PBS group, *n* = 6) (Figure 3(a)). The potentiated TRPA1 was decreased by administering auricular EA (107.7% ± 7.9%, *P* > .05 compared with KA group, *n* = 3) (Figure 3(a)), but not by administering EA at the ST36-ST37 acupoints (120.8% ± 14.2%,   *P* > .05 compared with EAR group, *n* = 6) (Figure 3(a)) or performing sham operation at the ST36-ST37 acupoints (116.9% ± 9.8%,   *P* > .05 compared with EAR group, *n* = 6) (Figure 3(a)). The TRPV4 antagonist has been proven to reduce epilepsy [24]. Therefore, we subsequently verified whether the TRPV4 protein levels changed after KA injection. The data used in this study revealed that the TRPV4 protein levels were unaltered after the KA injection (97.6% ± 3.0%,   *P* > .05, *n* = 6) (Figure 3(b)). Additionally, TRPV4 protein levels were unchanged after administering auricular and somatic EA at the ST36-ST37 acupoints (106.4% ± 5.2% and 98.0% ± 3.3%, resp., *P* > .05 compared with PBS group, *n* = 6) (Figure 3(a)). A similar result was obtained for the sham group (99.2% ± 5.3%,   *P* > .05 compared with PBS group, *n* = 6) (Figure 3(a)).

### 3.4. pPKC*α* and pERK1/2 Expressions Increased in the Hippocampus during an Epileptic Seizure

**Figure 4:**
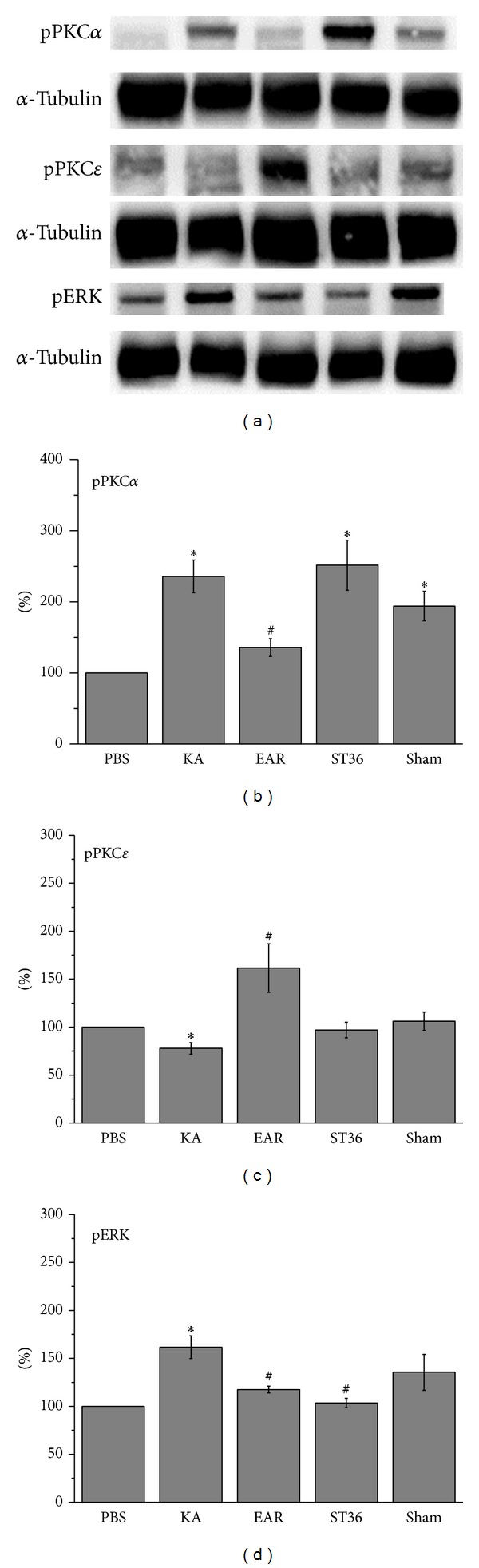
pPKC*α*, pPKC*ε*, and pERK protein levels. Hippocampus lysates were immunoreacted with specific pPKC*α*, pPKC*ε*, and pERK. (a) pPKC*α* and pERK, but not pPKC*ε*, increased with KA injection as compared with the PBS group. All results were analyzed and plotted as bar chart in (b), (c), and (d).

The significant change in pPKC isoforms that occurs during pilocarpine-induced epilepsy has been established [21, 22]. Therefore, we verified whether pPKC*α* and pPKC*ε* were involved in KA-induced epileptic seizures. The results indicated that pPKC*α* increased after KA injection (230.7% ± 22.9%,  *P* < .05 compared with PBS group, *n* = 6) (Figure 4(a)) and could be attenuated by administering auricular EA (135.7% ± 12.6%,  *P* < .05 compared with KA group, *n* = 6) (Figure 4(a)), but not by administering somatic EA (251.2% ± 35.1%,  *P* > .05,  *n* = 6) (Figure 4(a)). This phenomenon was not observed in the sham group (332.1% ± 47.2%,  *P* > .05,  *n* = 6) (Figure 4(a)). By contrast, pPKC*ε* decreased in the hippocampi of the KA-induced rats (77.9% ± 6.0%,  *P* < .05 compared with PBS group, *n* = 6) (Figure 4(a)). Potentiation was significantly reversed in the auricular EA group (161.6% ± 25.3%,  *P* < .05 compared with KA group, *n* = 6), but not in the somatic or sham groups (97.0% ± 8.1% and 106.1% ± 9.7%, resp., *n* = 6) (Figure 4(a)). In addition, we determined that the expression of pERK1/2 was similar to that of pPKC*α*. The pERK1/2 expression was augmented in the KA group (161.7% ± 11.8%,  *P* < .05 compared with PBS group, *n* = 6) and was decreased by administering auricular (117.5% ± 3.6%,  *P* < .05 compared with KA group, *n* = 6) and somatic EA (103.6% ± 4.9%,  *n* = 6) (Figure 4(a)). This phenomenon was not observed in the sham group (135.5% ± 18.2%,  *n* = 6) (Figure 4(a)).

## 4. Discussion

We first induced an epileptic seizure animal model by using a KA ip injection to investigate the effect of 2 Hz EA at the auricular and at ST36-ST37 acupoints. The results indicated that administering 2 Hz EA at the auricular and at ST36-ST37 can effectively reduce electrical discharge in the hippocampal CA1 areas of KA-treated rats. We also observed that the TRPA1, but not TRPV4, protein levels increased in the KA-treated rats. Furthermore, pPKC*α* and pERK1/2 increased. By contrast, pKC*ε* was reduced in rats during an epileptic seizure. EA administered at the auricular, but not at the ST36-ST37 acupoints, reversed the protein change in the hippocampus. Thus, we obtained novel evidence suggesting that EA administered at the ear can reduce epileptiform discharge accompanied by changes in the TRPA1, pPKC*α*, pPKC*ε*, and pERK1/2 signaling pathways.

A cooling temperature of less than 24°C is required to decrease neuronal function [43]. In addition, inhibiting experimental seizures requires a temperature of less than 24°C because complete cessation occurs at a temperature from <20°C to 22°C. Under this condition, several TRP channels were activated, which can influence seizures [43]. The results indicated that TRPA1, but not TRPV4, protein levels increased in the KA-induced epilepsy model. This phenomenon could be reversed in the EA at auricular group but not the ST36-ST37 or sham groups. During a seizure, increased TRPA1 protein levels may be responsible for cooling brain hyperactivity. In clinics, a high temperature has frequently been reported to trigger seizures. Because GABA_A_ inhibitory synaptic transmission is highly sensitive to temperature, decreasing the temperature serves as a convulsant drug [44].

Several PKC isoforms have been suggested to be involved in epileptogenesis based on a seizure model. Tang et al. reported that PKC*ε* decreased in the stratum granulosum during pilocarpine-induced epilepsy. After epileptic time was extended to 31 days, PKC*ε* expression disappeared in the inner molecular layer of the hippocampus during a seizure. PKC*ε* was suggested to participate in controlling inhibitory synaptic transmission [35]. The result of this study indicated that PKC*ε* protein levels were attenuated in KA-induced seizure rats and could be potentiated in the auricular group but not the ST36-ST37 or sham groups. Furthermore, PKC*α* demonstrated a dramatically different pattern in which PKC*α* increased in the KA-treated rats and could be reversed by administering auricular EA but not by ST36-ST37 EA or sham control. These results are similar to those described in previous publications, which have reported that PKC*α* increased in the stratum pyramidale of the CA3 area during pilocarpine-induced epilepsy [34]. In addition, we observed that pERK1/2 expression was augmented in the KA-induced seizure rats and reduced by administering auricular and somatic EA. These phenomena indicated the possible mechanisms that may be involved in EA-mediated antiepileptic curative effects.

## 5. Conclusion

In summary, we determined the effect of auricular and somatic EA on KA-induced seizure rats. The results suggest that the therapeutic effect was due to reduced hippocampal hyperactivity accompanied by the TRPA1, PKC*ε*, PKC*α*, and pERK1/2 signaling pathways. The related mechanisms could be reversed by administering auricular EA and could be partially reversed by ST36-ST37 EA. These novel findings are remarkable and compelling and reveal the crucial curative effects of auricular EA.
